# EHMTI-0285. Frontal thermography in healthy individuals: reliability of the method

**DOI:** 10.1186/1129-2377-15-S1-E39

**Published:** 2014-09-18

**Authors:** C Voiticovschi-Iosob, F Antonaci, C Fattore, E Rossi, M Bianchi, G Dalla Volta, G Nappi

**Affiliations:** 1 Headache Center, C. Mondino National Institute of Neurology Foundation IRCCS Dept. of Brain and Behavioral Sciences University of Pavia and State Medical and Pharmaceutical University “NicolaeTestemitanu”, Chisinau, Moldova; 2 Headache Center, C. Mondino National Institute of Neurology Foundation IRCCS Dept. of Brain and Behavioral Sciences University of Pavia, Pavia, Italy; 3 Clinical Trial Center and AntiepilepticDrugs, C. Mondino National Institute of Neurology Foundation IRCCS, Pavia, Italy; 4 Dipartimento di Elettronica Informazione e Bioingegneria, Politecnico di Milano, Milano, Italy; 5 U.O Neurologia, Istituto Clinico Città di Brescia, Brescia, Italy; 6 Headache Centre, C. Mondino National Institute of Neurology Foundation IRCCS Dept. of Brain and Behavioral Sciences University of Pavia, Pavia, Italy

## Introduction

Infrared Thermography (TH) is useful in making diagnoses and doing evaluations in the field of pain medicine.

## Methods

26 volunteers (20 females and 6 males) with a mean age of 32±10 years were examined. Seven volunteers had a history of low frequency migraine.TH has been assessed with an infrared thermal camera (model LT3, Zhejiang Dali Technology Co. Ldt) measuring the spatial distribution of the heat over the face. The image analysis evaluated the temperature in two target points (left (L) and right (R)) side) in the frontal polar sites. The measurements were taken in one session (N=26), 19 subjects underwent a second session at least one day apart. The Asymmetry Index (AI) (100 x (L side/Lside+R side)was also calculated. Concerning the first test session analysis of variance (ANOVA 1 way), intra-class correlation coefficient (ICC) and Pearson’s correlation coefficient were calculated. Measurements between two different days (T1 and T2 session) were evaluated with the ANOVA 2 way with replication.

## Results

The analysis of variance showed no significant difference between three consecutive measurements during the first session both on the R side (p=0.30)and the L side (p=0.32). Both R and LICC measurements showeda good reliability (0.55 and 0.66). TH values in healthy subjects showed no large asymmetry (49.98±0.22). ANOVA 2 way did not revealintraindividual variations between the first and second testing session on separate days.

## Conclusion

TH measurements were rather symmetrical and reproducible on both sides. TH could be a reliable method for the evaluation of localized/lateralized pain syndrome.

No conflict of interest.

